# A machine learning approach using ^18^F-FDG PET and enhanced CT scan-based radiomics combined with clinical model to predict pathological complete response in ESCC patients after neoadjuvant chemoradiotherapy and anti-PD-1 inhibitors

**DOI:** 10.3389/fimmu.2024.1351750

**Published:** 2024-01-30

**Authors:** Wei-Xiang Qi, Shuyan Li, Jifeng Xiao, Huan Li, Jiayi Chen, Shengguang Zhao

**Affiliations:** ^1^Department of Radiation Oncology, Ruijin Hospital, Shanghai Jiaotong University School of Medicine, Shanghai, China; ^2^Real Time Lab, Shenzhen United Imaging Research Institute of Innovative Medical Equipment, Shenzhen, China

**Keywords:** radiomics, neoadjuvant chemoradiotherapy, esophageal squamous cell carcinoma, pathological complete response, immune checkpoint inhibitor

## Abstract

**Background:**

We aim to evaluate the value of an integrated multimodal radiomics with machine learning model to predict the pathological complete response (pCR) of primary tumor in a prospective cohort of esophageal squamous cell carcinoma (ESCC) treated with neoadjuvant chemoradiotherapy (nCRT) and anti-PD-1 inhibitors.

**Materials and methods:**

Clinical information of 126 ESCC patients were included for analysis. Radiomics features were extracted from ^18^F-FDG PET and enhanced plan CT images. Four machine learning algorithms, including SVM (Support Vector Machine), Random Forest (RF), and eXtreme Gradient Boosting (XGB) and logistic regression (LR), were applied using k-fold cross-validation to predict pCR after nCRT. The predictive ability of the models was assessed using receiver operating characteristics (ROC) curve analysis.

**Results:**

A total of 842 features were extracted. Among the four machine learning algorithms, SVM achieved the most promising performance on the test set for PET(AUC:0.775), CT (AUC:0.710) and clinical model (AUC:0.722). For all combinations of various modalities-based models, the combination model of ^18^ F-FDG PET, CT and clinical features with SVM machine learning had the highest AUC of 0.852 in the test set when compared to single-modality models in various algorithms. The other combined models had AUC ranged 0.716 to 0.775.

**Conclusion:**

Machine learning models utilizing radiomics features from ^18^F-FDG PET and enhanced plan CT exhibit promising performance in predicting pCR in ESCC after nCRT and anti-PD-1 inhibitors. The fusion of features from multiple modalities radiomics and clinical features enhances the better predictive performance compared to using a single modality alone.

## Introduction

Esophageal cancer is one of the world’s most common diagnosed gastrointestinal cancers and cancer-related death (1). In China, esophageal squamous cell carcinoma (ESCC) is the major histological type of esophageal carcinoma (2). Since the publication of CROSS (3, 4) and NEOCRTEC 5010 (5) phase III trials, the standardized treatment option for locally advanced ESCC is a neoadjuvant chemoradiotherapy followed by esophagectomy. However, esophagectomy is a highly invasive surgery. Although the mortality followed by esophageal cancer surgery has been reduced from 30% in pre-1980 to less than 5% in recent years, occurrence of operation morbidity remains high than 50%, which significantly impact quality life of EC patients (6, 7). Radiation plays an important role in organ preservation for EC patients, with a watch and wait strategy enabling surgery to be avoided in patients who are refusing surgery. In this contest, a non-surgical, “organ preservation” followed by close follow-up strategy is recommended for EC patients with a complete clinical response after nCRT. However, in order to safely provide organ preservation without increasing the risk of post-treatment recurrence, an accurate prediction of the tumor response after nCRT in ESCC patients is clearly required.

Recently, radiomics, which is defined as the process of converting medical images into high-dimensional, mineable, and quantitative imaging features via high-throughput data extraction algorithms, might improves the predicting accuracy of survival outcomes. However, most established radiomics models have not been used for routine clinical treatment due to some limitations, including inconsistent standards, heterogeneous methods, and lack of quality control or external validation. Therefore, the clinical application of radiomics to guide cancer treatment strategies is still needed further research and exploration. Prior to the present study, Wang J. et al (8) demonstrated that machine learning models based on pretreatment CT image radiomic features combined with clinical model could accurately predict response to therapy of esophageal squamous cell carcinoma patients after nCRT with an AUC of 0.891 (95% CI: 0.823–0.950) in the testing set. Frood R. et al (9) found that pre-treatment FDG PET-CT-based models could predict the survival outcomes of ESCC patients with a training c-index of 0.7 and an external testing c-index of 0.7. However, it is still unknown that whether multimodal radiomics features could be more predictive relative to single-dimensional model. In addition, which machine learning algorithms would be the optimal method to identify the radiomics features remain unknown. More recently, immune checkpoint inhibitors (ICIs) had been investigated in different stages of clinical trials (10, 11). The phase III Checkmate 648 trial (12) showed that both first-line treatment with nivolumab plus chemotherapy (fluorouracil + cisplatin) resulted in significantly longer overall survival than chemotherapy alone in patients with advanced esophageal squamous-cell carcinoma (13.2 vs. 10.7 months, HR 0.74 [0.58–0.96], p = 0.002). KEYNOTE-590 (13) showed that pembrolizumab plus chemotherapy was superior to chemotherapy alone as first-line treatment for locally advanced oesophageal cancer in all randomised patients (12·4 months vs 9·8 months; 0·73 [0·62-0·86]; p<0·0001). Additionally, other ICIs including camrelizumab, toripalimab and sintilimab also improved median OS and PFS in overall populations in the clinical trials of ESCORT-1st (14), JUPITER-06 (15), ORIENT-15 (16). Based on these published trials, PD-1/PD-L1 inhibitors had become the standard of care for the treatment of metastatic esophageal cancer (12, 13, 17, 18).

Additionally, multiple clinical trials had been performed to investigate the efficacy and toxicities of ICIs as neoadjuvant treatment for ESCC (19, 20). Zhu M. et al (19) found that the addition of pembrolizumab to nCRT in gastroesophageal junction cancer adenocarcinoma could improve pCR in patients with PD-L1 CPS ≥ 10 (50% vs. 13.6%, p = 0.046), compared with those with CPS < 10. The PERFECT trial conducted by van den Ende et al. (21) demonstrated that the combination atezolizumab with CRT showed a pCR rate of 25% but without survival benefit for resectable esophageal adenocarcinoma. For locally advanced ESCC patients, the addition of ICIs to neoadjuvant chemotherapy showed pCR between 16.7-35.3% (22, 23).PALACE-1 trial conducted by our institute showed that the combination of pembrolizumab with nCRT in ESCC patients achieved a pCR of 55.6% in ESCC patients (24). Therefore, we perform the present study to evaluate the value of an integrated multimodal radiomics with machine learning combined model to predict the pathological complete response (pCR) of primary tumor in a prospective cohort of esophageal squamous cell carcinoma (ESCC) treated with neoadjuvant chemoradiotherapy (nCRT) and anti-PD-1 inhibitors. Additionally, we compare the predictive ability of the models based on four different machine learning algorithms.

## Materials and methods

### Study papulation

The study was designed as a retrospective design from our prospective trials (NCT NCT04435197, NCT04435197, NCT04513418, NCT03990532) (25–27), and all included patients were treated with nCRT and PD-1 inhibitors followed by esophagectomy at Ruijin Hospital, Shanghai Jiao Tong university school of medicine, between Jan 2019 and July 2023. All included patients received standardized neoadjuvant chemoradiotherapy. The chemotherapy regimen consisted of carboplatin (area under the curve of 2 mg/mL per min) and paclitaxel or nab-paclitaxel (50 mg/m2 of body surface area), which were administered intravenously on days 1, 8, 15, 22. Concurrent radiotherapy was performed on day 1 of chemotherapy with a total dose of 41.4 Gy in 23 fractions, on five fractions per week (28). Pembrolizumab was given on days 1 and 22 of the neoadjuvant therapy intravenously at 200mg. Since the publication of CheckMate 577 trial (29), adjuvant nivolumab among patients with resected esophageal or gastroesophageal junction cancer who had received nCRT could improve disease-free survival. Therefore, adjuvant ICIs was recommended for ESCC patients who did not archive pCR after neoadjuvant CRT combined with pembrolizumab. Finally, a total of 126 patients with histologically proven ESCC with pre-treatment ^18^ FDG PET/CT and enhanced plan CT images were included for analysis. Data regarding surgical procedures, neoadjuvant therapy, and potential confounding clinical and demographic data [(sex, age, body mass index, medical history, smoking status, alcohol use, location of tumor, radiotherapy modality, location of anastomosis, clinical TNM stage, pathological TNM, baseline white blood cell (WBC), lymphocytes count (LY), neutrophil, Monocyte count] were manually extracted.

### Workflow of treatment response prediction

**Figure 1:**
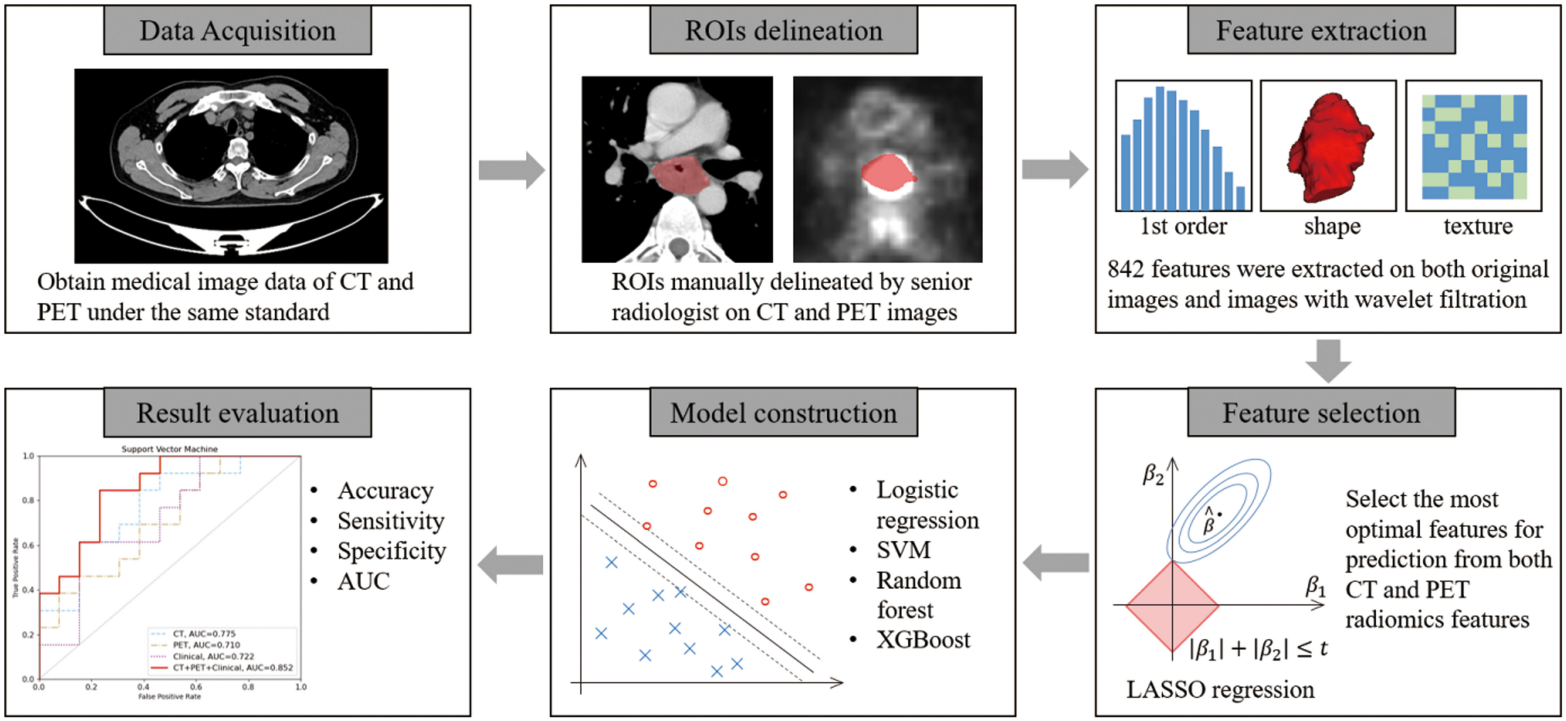
Treatment response prediction workflow.

The workflow of treatment response prediction in this study is illustrated in **Figure 1**. Each input case consists of a contrast-enhanced plan CT and PET scan, along with the corresponding GTV delineation. A total of 842 features were extracted, and the top 10 features with the highest occurrences in LASSO were selected for both CT and PET features, respectively. In this study, we employed four different machine learning algorithms, namely Support Vector Machine, Logistic Regression, Random Forest, and XGBoost, to predict treatment response. Each algorithm was tested on CT features, PET features, and CT-PET fused features. During the evaluation stage, we calculated the accuracy, sensitivity, specificity, and the AUC value of each model to compare their performance.

### Radiomics feature extraction

The extraction of radiomics features was performed automatically using the open-source Python library PyRadiomics. Features were computed based on the radiologist-drawn ROIs on both CT and PET images. The computed features include first-order based features, 3D morphology-based features, and texture analysis features. The texture analysis features consist of Gray Level Cooccurence Matrix (GLCM), Gray Level Run Length Matrix (GLRLM), Gray Level Size Zone Matrix (GLSZM), Gray Level Dependence Matrix (GLDM), and Neighboring Gray Tone Difference Matrix (HGTDM). All the aforementioned features were extracted from both the original images and the images with wavelet filtration. The wavelet filter comprises two types: low-pass (L) and high-pass (H). In this study, the radiomics features were extracted from the 3D images, thus the wavelet features were computed along the x, y, and z directions. The features with wavelet filtration can be categorized into eight categories: wavelet-HLL, wavelet-LHL, wavelet-LHH, wavelet-LLH, wavelet-HLH, wavelet-HHH, wavelet-HHL, and wavelet-LLL.

### Feature selection

To mitigate the Curse of Dimensionality and address potential adverse effects of data distribution, feature selection was conducted to identify the most robust features for machine learning tasks. We employed the variance threshold selection and the least absolute shrinkage and selection operator (LASSO) logistic regression analysis, utilizing the open-source Python library Scikit-learn, to select the best features for prediction. During the feature selection stage, we initially applied a variance threshold selection with a threshold of 1 to the input 842 features, resulting in the selection of approximately 200 features. Subsequently, LASSO regression was performed on the remaining features to identify the most significant ones, employing 5-fold cross validation with α = 1e-2. The feature selection process was repeated 100 times, and the optimal features with the highest occurrences were selected as the input for the machine learning model. Furthermore, features with higher weights were considered to have a greater impact on the task and were retained by the LASSO regression.

### Model construction and evaluation

In this study, we utilized several widely-used machine learning algorithms for the prediction task, including logistic regression, SVM (Support Vector Machine), Random Forest, and XGBoost (eXtreme Gradient Boosting), all implemented using the Scikit-learn library.

#### Logistic regression

Logistic Regression is a type of generalized linear model, represented by the model form $w^{\prime }\text{x}+\text{b}$ . The model’s domain is defined as [-∞, +∞], but its output values are limited to two categories: {0, 1} (30). Consequently, the classification of input data x is achieved by mapping the continuous range of real numbers to a finite number of points.

#### Support vector machine

Support Vector Machine (SVM) is a type of generalized linear classifier that performs binary classification through supervised learning. It determines the decision boundary by identifying the maximum margin hyperplane based on the training samples. In cases where the classification task involves non-linear data, kernel methods can be employed to map the input data into a high-dimensional linear space, enabling effective classification.

#### Radom forest

Random Forest is an ensemble learning method that constructs a forest consisting of multiple decision trees in a random manner. It can be seen as a variant of bagging, where decision trees are employed as the underlying models. Random Forest randomly selects subsets of features and training data, and then aggregates the predictions from these individual trees to make the final prediction. The prediction is determined by selecting the label with the highest frequency among the predictions of the constituent trees.

#### XGBoost

XGBoost, short for eXtreme Gradient Boosting, is a powerful machine learning algorithm known for its exceptional performance in various tasks. It is an optimized implementation of the gradient boosting framework that combines multiple weak learners to create a strong predictive model. XGBoost utilizes a boosting technique, where weak learners, typically decision trees, are sequentially added to the ensemble. Each new tree is trained to correct the errors made by the previous ones. This iterative process enables XGBoost to learn complex patterns and make accurate predictions.

We applied all the aforementioned machine learning methods to construct the prediction model and compared their performance. Thirteen patients were randomly assigned to the test cohort, while the remaining fifty-two patients were used for training. The features selected by LASSO regression were utilized as inputs for the model, and the model’s output was classified as either “pCR” or “non-pCR”. To determine the optimal parameters, the training stage employed the 5-fold cross-validation method. In this study, accuracy, sensitivity, and specificity were chosen as the evaluation metrics to assess the model’s performance on the testing cohort. Additionally, the predictive performance of the model was evaluated using the area under the curve (AUC) of the receiver operating characteristic (ROC) curve.

### Model hyperparameters:

SVM:

CT model: (C=10, cache_size=2000, class_weight=‘balanced’, coef0 = 0.0, decision_function_shape=‘ovr’, degree=3, gamma=‘auto’, kernel=‘rbf’, max_iter=30000, probability=True, random_state=None, shrinking=True, tol=0.001, verbose=False)

PET model: (C=0.08, cache_size=2000, class_weight=‘balanced’, coef0 = 0.0, decision_function_shape=‘ovr’, degree=3, gamma=‘auto’, kernel=‘linear’, max_iter=30000, probability=True, random_state=None, shrinking=True, tol=0.001, verbose=False)

Clinical model: (C=25, cache_size=2000, class_weight=‘balanced’, coef0 = 0.0, decision_function_shape=‘ovr’, degree=3, gamma=‘auto’, kernel=‘rbf’, max_iter=30000, probability=True, random_state=None, shrinking=True, tol=0.001, verbose=False)

Logistic regression:

CT model: (C=0.15, class_weight=‘balanced’, penalty=‘l2’, solver=‘liblinear’)

PET model: (C=50, class_weight=‘balanced’, penalty=‘l2’, solver=‘liblinear’)

Clinical model: (C=10, class_weight=‘balanced’, penalty=‘l1’, solver=‘liblinear’)

Random forest:

CT model: (max_depth=3, n_estimators=150, class_?>weight=‘balanced’, max_features=‘auto’, criterion=‘gini’, min_samples_leaf=1, min_samples_split=7, bootstrap=True)

PET model: (max_depth=3, n_estimators=150, class_weight=‘balanced’, max_features=‘auto’, criterion=‘gini’, min_samples_leaf=3, min_samples_split=7, bootstrap=True)

Clinical model: (max_depth=3, n_estimators=150, class_weight=‘balanced’, max_features=‘auto’, criterion=‘gini’, min_samples_leaf=1, min_samples_split=7, bootstrap=True)

XGBoost

CT model: (eta=1e-1, gamma=0.1, max_depth=2, min_child_weight=0.5, subsample=0.9, colsample_bytree=0.1, alpha=1e-3, scale_pos_weight=1)

PET model: (eta=1e-4, gamma=0.1, max_depth=2, min_child_weight=0.5, subsample=0.9, colsample_bytree=0.1, alpha=1e-3, scale_pos_weight=1)

Clinical model: (eta=1e-3, gamma=0.1, max_depth=2, min_child_weight=0.5, subsample=0.9, colsample_bytree=0.1, alpha=1e-5, scale_pos_weight=1)

### Feature fusion

The aforementioned machine learning models were constructed and trained using features from a single modality using CT, PET and clinical features. Additionally, a fusion process was conducted at the backend by combining the output probabilities and selected thresholds from both the CT feature model, the PET feature model and the clinical feature model. This fusion process involved weighting the probabilities and summing them to obtain the final output value. Subsequently, classification was performed using the weighted threshold to determine the ultimate decision and classification result based on the fusion. It is important to note that the fusion process is currently limited to models utilizing the same machine learning algorithm. Different machine learning models are not fused together. Ultimately, we will compare the performance of models using only CT features, models using only PET features, models using only clinical features, and the fused model incorporating CT+PET+clinical features.

### Statistical analysis

In this study, we utilized the open-source Python library PyRadiomics to extract radiomics features. We selected the most optimal features for prediction using LASSO regression implemented in scikit-learn library (version 1.0.2). Furthermore, we trained SVM, logistic regression, and random forest models using scikit-learn, and xgboost model using the xgboost library. During the evaluation stage, we employed matplotlib to plot the ROC curve and utilized scikit-learn to compute the AUC value for each model. Performance of models was quantified as AUC, sensitivity and specificity. Coefficient of logical regression was applied for feature importance ranking. Moreover, we utilized Scipy for statistical analysis. Continuous variables were described using means and standard deviations, and categorical variables were demonstrated with percentages. A two-sided *p* < 0.05 was regarded as statistical difference.

## Results

### Baseline characteristics

A total of 126 patients were finally included for analysis in the present study. Of them, 83 patients (65.9%) present with stage III and 32 patients (25.4%) with stage IVA. The median time from completion of nCRT to surgery were 42 days (range:14-89 days). The median tumor length was 5cm (range: 1-15cm). A total of 66 ESCC patients (52.4%) achieved primary tumor pathological complete response (pCR). The baseline characteristics of include patients were listed **Table 1**.

**Table 1:** Baseline characteristics of included patients.

Characteristics	Level	Statistic
**Gender**	Female	19
	Male	107
**Age at diagnosis**	Years (median, range)	66(39-80)
**Clinical T stage**	T1-2	15
	T3-4	111
**Clinical N stage**	N0-1	45
	N2-3	81
**Clinical stage**	II	11
	III	83
	IV	32
**Time from completion of NCRT to surgery**	Days (median, range)	42 days (14-89 days)
**BMI**	Median, range	22.43(17.13-30.47)
**Primary tumor pCR**	Yes	66
	No	60
**Tumor length**	cm (median, range)	5(1-15)
**Smoking status**	Yes	81
	No	45
**Drinking status**	Yes	77
	No	49
**Tumor location**	Upper thoracic	22
	Middle thoracic	35
	Low thoracic	69
**WBC**	Median, range	5.92(3.1-16.6)*10^9^/L
**LY**	Median, range	1.41(0.3-2.75)*10^9^/L
**Monocyte count**	Median, range	0.43(0.15-7.3)*10^9^/L
**neutrophil**	Median, range	3.71(1.5-13.6)*10^9^/L
**NLR**	Median, range	2.64(1.04-11.13)
**PLR**	Median, range	142.24(61.2-743.4)
**LMR**	Median, range	3.37(0.21-10.67)
**PIV**	Median, range	220.51(63.83-18598.45)

BMI, body mass index; LMR, lymphocyte-to-monocyte ratio; NLR, neutrophil-to-lymphocyte ratio; pCR, pathological complete response; PIV, pan-immune inflammation value; PLR, platelet-to-lymphocyte ratio; LY, lymphocyte count; WBC, white blood cell; NSE.

### Feature selection

In this study, a total of 100 cases were randomly divided into the training cohort, while the remaining 26 cases were allocated to the test cohort. The LASSO method was employed to select the optimal features for prediction from the extracted features. During the feature extraction stage, a comprehensive set of 1684 features was computed for each patient from the input images. Half of these features were computed from CT scans, while the other half were derived from PET scans. Feature selection was performed separately on the CT and PET features, resulting in the selection of 20 features from CT and 30 features from PET. Among the selected features, 40 were derived from wavelet filtering, while 10 features originated from the original image features. All of the selected features were presented in **Table 2** for reference.

**Table 2:** Finalized features and clinical characteristics identified and incorporated in the model construction

CT feature names	P-value
*wavelet-LHL_glszm_LowGrayLevelZoneEmphasis*	0.0049
*wavelet-LHL_glszm_HighGrayLevelZoneEmphasis*	0.0049
*wavelet-LLH_glcm_Idm*	0.0027
*wavelet-LHL_glrlm_LongRunHighGrayLevelEmphasis*	0.0088
*wavelet-LHL_gldm_LargeDependenceHighGrayLevelEmphasis*	0.0043
*wavelet-LHL_glcm_SumEntropy*	0.0139
*wavelet-LHL_glrlm_LowGrayLevelRunEmphasis*	0.0115
*wavelet-LLH_glcm_ClusterProminence*	0.0062
*wavelet-LLL_glrlm_RunLengthNonUniformityNormalized*	0.0118
*original_gldm_DependenceEntropy* *wavelet-LHL_firstorder_Entropy* *original_glrlm_RunLengthNonUniformityNormalized* *wavelet-LLH_glrlm_LowGrayLevelRunEmphasis* *wavelet-HLH_glcm_SumSquares* *original_glcm_DifferenceEntropy* *wavelet-LLH_ngtdm_Contrast* *wavelet-LLH_glcm_Correlation* *wavelet-LLH_ngtdm_Complexity* *wavelet-HLH_firstorder_Entropy* *wavelet-LHL_firstorder_Mean*	0.0046 0.0063 0.0111 0.0148 0.0118 0.0209 0.0017 0.0047 0.0017 0.0148 0.0226
PET feature names	P-value
*wavelet-HHH_glszm_SizeZoneNonUniformity*	0.0146
*wavelet-HHH_glszm_GrayLevelNonUniformity*	0.0044
*wavelet-HHH_glszm_SmallAreaLowGrayLevelEmphasis* *original_shape_Sphericity* *wavelet-HLH_glszm_ZoneEntropy* *wavelet-HLH_glszm_GrayLevelNonUniformity* *wavelet-LHH_glcm_SumEntropy* *wavelet-HLH_glszm_HighGrayLevelZoneEmphasis* *wavelet-LHL_firstorder_Entropy* *original_glrlm_ShortRunLowGrayLevelEmphasis* *wavelet-LHL_glcm_SumEntropy* *wavelet-HLH_firstorder_Mean* *wavelet-LLH_glszm_ZoneEntropy* *wavelet-HHH_glszm_ZoneEntropy* *wavelet-LHL_glszm_SmallAreaLowGrayLevelEmphasis* *wavelet-HHL_glszm_LargeAreaHighGrayLevelEmphasis* *wavelet-LHL_gldm_DependenceNonUniformity* *wavelet-LLL_glszm_SmallAreaEmphasis* *wavelet-LHL_glszm_LargeAreaHighGrayLevelEmphasis* *wavelet-HLH_glszm_SizeZoneNonUniformityNormalized* *wavelet-LHL_gldm_DependenceNonUniformityNormalized* *wavelet-LHH_glcm_JointAverage* *original_shape_SurfaceArea* *wavelet-LLH_gldm_DependenceEntropy* *original_glszm_ZonePercentage* *original_gldm_GrayLevelNonUniformity* *original_glszm_ZoneVariance * *wavelet-LHL_glszm_SmallAreaEmphasis* *original_shape_Maximum2DDiameterSlice* *wavelet-HHL_glszm_LowGrayLevelZoneEmphasis*	0.0424 0.0353 0.0270 0.0299 0.0864 0.1032 0.0852 0.0923 0.0712 0.0822 0.1068 0.0799 0.1220 0.0685 0.0336 0.1135 0.0591 0.1071 0.0876 0.1047 0.0387 0.1088 0.0829 0.0832 0.1099 0.1549 0.0883 0.2005
Clinical Features	P-value
*tumor length*	0.0959
*BMI* *cStage* *time interval* *LY* *smoking status* *cT* *NSE* *cN* *NLR*	0.2735 0.5752 0.3496 0.5669 0.7571 0.6823 0.0789 0.8733 0.1345

### Performance of four machine learning radiomic and clinical-based models

**Figure 2:**
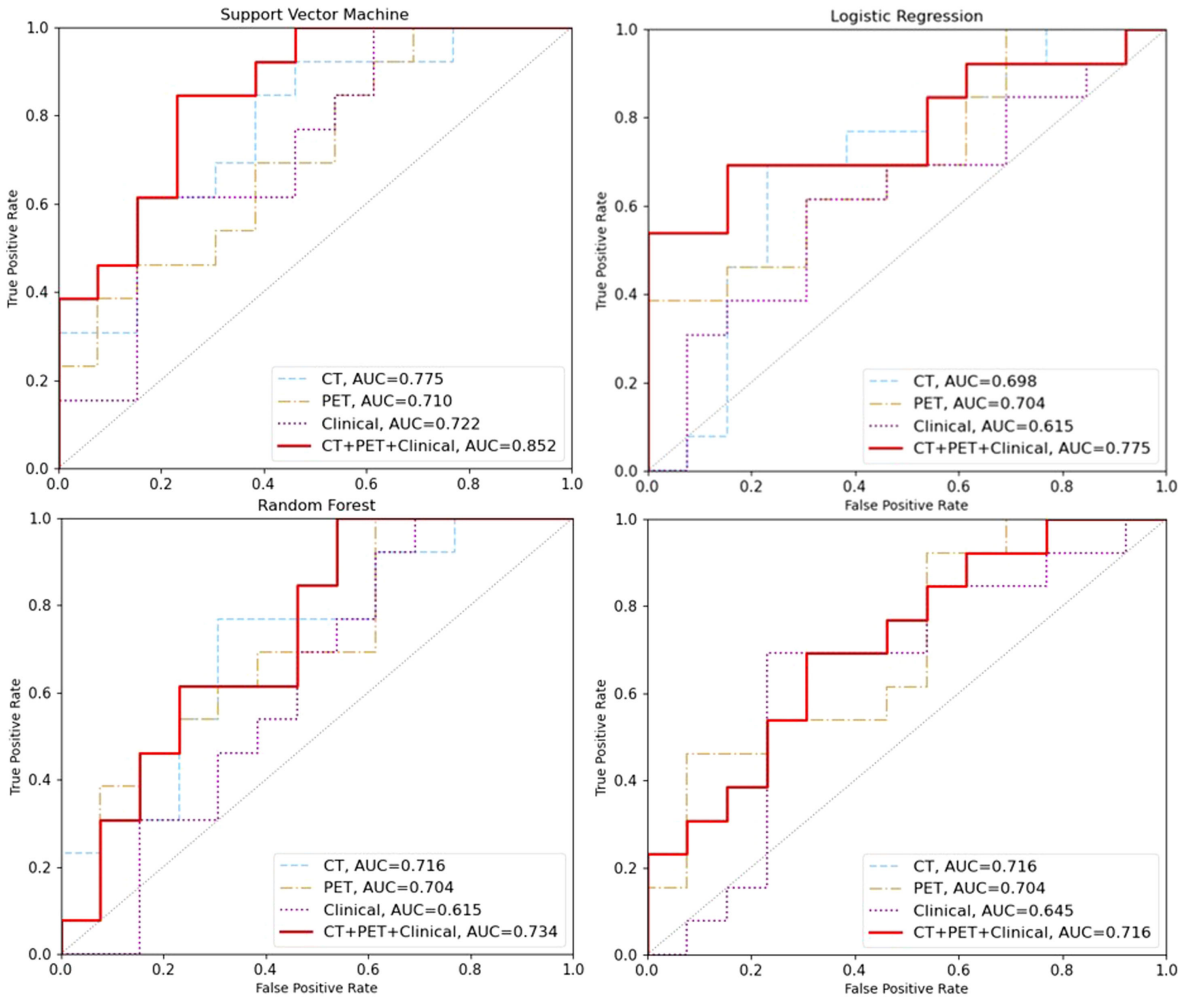
The area under the receiver operating characteristic curve (AUC) was used to evaluate the performance of different machine learning models on the test set. The AUC values for the models based on CT features, PET features, clinical features and CT+PET+clinical fused features are represented by the blue, brown, purple and red lines, respectively.

The results of the machine learning models were presented in **Table 3**. Four different models were implemented: Support Vector Machine (SVM), Logistic Regression (LR), Random Forest (RF), and XGBoost (XGB). The optimal hyper parameters for each model were tuned using the validation set, and their performance was evaluated on the test set. Each model was trained and tested using three different feature sets: CT features, PET features, clinical features. Our experimental results indicate that SVM achieved the most promising performance with AUC of 0.775 for CT and 0.710 for PET when compared to LR, RF and XGB models (AUC for CT: 0.698-0.716; AUC for PET 0.704, **Figure 2**). For clinical model, machine learning using SVM remained the most promising performance (AUC 0.772), when compared other machine learning methods (AUC ranges from 0.615-0.645).

**Table 3:** The prediction performance of different machine learning models, including Support Vector Machine (SVM), Logistic Regression (LR), Random Forest, and XGBoost, was evaluated.

SVM	Accuracy		Sensitivity		Specificity		AUC value	
	Train	Test	Train	Test	Train	Test	Train	Test
**CT**	0.950	0.692	0.981	0.846	0.917	0.538	0.991	0.775
**PET**	0.760	0.615	0.750	0.462	0.771	0.769	0.826	0.710
**Clinical**	0.970	0.715	0.942	0.615	0.938	0.615	0.996	0.722
**Fused**	0.970	0.808	0.990	0.846	0.938	0.769	0.997	0.852
LR	Accuracy		Sensitivity		Specificity		AUC value	
	Train	Test	Train	Test	Train	Test	Train	Test
**CT**	0.700	0.654	0.808	0.769	0.583	0.538	0.768	0.698
**PET**	0.760	0.615	0.769	0.462	0.750	0.769	0.846	0.704
**Clinical**	0.610	0.538	0.596	0.385	0.625	0.692	0.662	0.615
**Fused**	0.820	0.654	0.808	0.538	0.833	0.769	0.897	0.775
RF	Accuracy		Sensitivity		Specificity		AUC value	
	Train	Test	Train	Test	Train	Test	Train	Test
**CT**	0.840	0.654	0.885	0.769	0.792	0.538	0.932	0.716
**PET**	0.900	0.654	0.904	0.462	0.896	0.846	0.967	0.704
**Clinical**	0.780	0.577	0.865	0.385	0.688	0.769	0.871	0.615
**Fused**	0.950	0.654	0.962	0.846	0.938	0.462	0.987	0.734
XGBoost	Accuracy		Sensitivity		Specificity		AUC value	
	Train	Test	Train	Test	Train	Test	Train	Test
**CT**	0.930	0.654	0.962	0.615	0.896	0.692	0.989	0.716
**PET**	0.810	0.538	0.885	0.462	0.729	0.615	0.915	0.704
**Clinical**	0.730	0.654	0.827	0.538	0.625	0.769	0.803	0.645
**Fused**	0.940	0.654	0.962	0.615	0.917	0.692	0.989	0.716

Each model was tested using three different feature sets: CT features, PET features, and CT+PET fused features. SVM, Support Vector Machine; LR, Logistic Regression; RF, Random Forest.

### Performance of combined models in predicting pCR

In addition, we also investigate the predictive value of combined models trained on CT+PET+clincial fused features. Our result showed that SVM machine learning exhibited significantly higher results compared to those trained solely on CT or PET features in the training set (AUC=0.997) and in the testing set (AUC=0.852, 95%CI: 0.824-0.876, **Figure 2**). This demonstrates the potential of enhancing performance through muti-modalities feature fusion.

## Discussion

Until now, nCRT followed by esophagectomy remains the standard treatment for potentially curable ESCC patients. However, approximately 40% of these patients would archive pCR, with an estimated 5-year survival of 70-80% (3, 5). In our PALACE-1 trial, the addition of pembrolizumab to nCRT could improve the pCR to 55.5% with acceptable toxicities, which suggested that the combination of ICIs with nCRT could be a novel treatment option for locally advanced ESCC. On the one hand, surgery likely contributed limited benefit for those favorable ESCC patients, and active surveillance, which had already been applied in patients with rectal cancer, could be considered as an alternative treatment option for ESCC patients with a clinically complete response (cCR) after nCRT. On the other hand, for those who do not respond to nCRT, earlies surgical intervention could minimize the nCRT-related toxicities. As a result, it is critically important to develop a preoperative, non-invasive approach to exactly predict pCR for ESCC treated with nCRT.

Prior to the present study, several studies had been performed to establish pCR predicting models by using clinical characteristics, imaging features, or biological markers. Hu Y. et al (31) trained model with features extracted from ResNet50 to predict the pCR with an AUC and accuracy of 0.805 (95% CI, 0.696-0.913) and 77.1% (65.6%-86.3%) in the testing cohort. Zhang M. et al (32) compared pre- and post-treatment CT-based radiomics and deep learning features for predicting pCR in patients with ESCC receiving nCRT using three machine learning classifiers and found that the XGBoost-based radiomics signature performed well. And the authors incorporated radscores and hematological biomarkers into pCR predicting model with AUCs of 0.857 in the testing set. More recently, Wang J. et al (8) built two machine learning models for predicting primary tumor CR and total pCR of ESCC patients who underwent nCRT with an AUC of 0.891 and 0.814 in the testing set. However, most of these predicting models were trained based on retrospective ESCC cohorts treated with nCRT alone, Limited radiomics model could be obtained for predicting pCR of ESCC treated with NCRT and PD-1 inhibitors. As far as we know, our study was the first to build a multiple modalities radiomics combined with clinical models based on four machine learning methods in a prospective cohort of ESCC patients treated with standardized nCRT and PD-1 inhibitors followed by surgery, which would contribute to greater generalisability of the present model. Based on our analysis, machine learning using SVM achieved the most promising performance when compared to LR, RF and XGB models. Furthermore, the models trained on CT+PET+clincial fused features by using SVM machine learning exhibited significantly higher results compared to those trained solely on CT or PET features in the training set (AUC=0.997) and in the testing set (AUC=0.852, 95%CI: 0.824-0.876). However, further studies were still needed to externally validate the combined model among the ESCC patient population treated with standardized nCRT and PD-1 inhibitors.

As for clinical model, a total of ten keys clinical features were identified to establish clinical model with an AUC of 0.722 in the test set. In the clinical model, patients with higher TNM staging or longer tumor length indicated increased tumor burdens, and elevated tumor burdens correlated with low probability of pCR. Several studies had demonstrated that longer interval between nCRT and surgery was associated with improved pathological response (33, 34). In consistent with those findings, time interval from complete of nCRT and surgery in the present study was another indicator which could impact the probability of archiving pCR. Additionally, we also found that baseline blood biomarkers lymphocytes count and NLR were associated with improved pathological response. In our previous study, we had demonstrated that pretreatment lymphocytes count was an independent predictor for achieving pCR and favorable outcomes of ESCC treated with neo-CRT and pembrolizumab (28), while NLR had been reported to be associated with pCR in ESCC patients in several studies (35–37). Some other clinicopathologic factors such as BMI and smoking status had also been associated with pCR. Based on these clinical features, we established a predictive clinical model, which was important and should be incorporating into radiomic features during training model.

The present study had the following strengths: firstly, we used multimodality including pretreatment enhanced plan CT and 18-FDG PET images for machine learning to improve the model reliability. In addition, we compared the predictive value of four different machine learning models. Secondly, all of the included patients were identified from a prospective cohort and all patients were treated with standardized nCRT and PD-1 inhibitors, this was the first study to assess the predictive role of radiomics in ESCC treated with nCRT and PD-1 inhibitors. Additionally, the radiotherapy regimen was 41.4Gy/23Fx, and concurrent chemotherapy regimen was same and consisted of carboplatin (area under the curve of 2 mg/mL per min) and paclitaxel or nab-paclitaxel (50 mg/m2 of body surface area). which would reduce the impact of heterogeneity from nCRT on the radiomics models.

However, this study had some limitations. Firstly, long-term survival outcomes of ESCC after nCRT combined with ICIs could not be available, thus whether our established model could predict the overall survival of ESCC patients remain unknown, although pCR was a critical factor in predicting long-term survival of patients with esophageal cancer after preoperative therapy (38). Secondly, this study was single center study with a relatively small patient population and absence of an external groups for validation, further multicentric researches were still recommended to confirm our proposed pCR model. Finally, there was lack of standardized assessment for lymph node metastasis, and it was difficult to delineate the metastatic lymph nodes from benign lymph nodes, especially for small lymph nodes. Therefore, imaging features that reflected metastatic lymph node characteristics may be not accurate enough, we thus only assessed the value of an integrated multimodal radiomics with machine learning combined model to predict the pCR of primary tumor after nCRT in ESCC patients.

## Conclusion

In conclusion, our findings indicated that machine learning algorithms using SVM achieved the most promising performance when compared to LR, RF and XGB models. In addition, we found that machine learning models utilizing integrated multimodal PET and CT-images combined with clinical features exhibited promising performance in predicting pCR of ESCC treated with standardized nCRT and PD-1 inhibitors, which would provide an exactly preoperative, non-invasive approach to predict pCR for ESCC and guidance for further precision treatment.

## Data availability statement

The raw data supporting the conclusions of this article will be made available by the authors, without undue reservation.

## Ethics statement

The studies involving humans were approved by Ethics Committee of Ruijin hospital, Shanghai Jiao Tong University School of Medicine. The studies were conducted in accordance with the local legislation and institutional requirements. The participants provided their written informed consent to participate in this study.

## Author contributions

W-XQ: Conceptualization, Formal analysis, Funding acquisition, Methodology, Project administration, Writing – original draft. SL: Data curation, Funding acquisition, Investigation, Project administration, Validation, Writing – original draft. JX: Methodology, Resources, Writing – original draft, Formal analysis, Project administration. HL: Data curation, Investigation, Methodology, Visualization, Writing – original draft. JC: Data curation, Investigation, Funding acquisition, Project administration, Validation, Writing – review & editing. SZ: Validation, Conceptualization, Methodology, Resources, Writing – original draft.
